# Lesotho's Minimum PMTCT Package: lessons learned for combating vertical HIV transmission using co-packaged medicines

**DOI:** 10.7448/IAS.15.2.17326

**Published:** 2012-12-26

**Authors:** Lotus McDougal, Mpolai M Moteetee, Florence Mohai, Malisebo Mphale, Binod Mahanty, Blandinah Motaung, Victor Ankrah, Makaria Reynolds, Kenneth Legins, Appolinaire Tiam, Chewe Luo, Craig McClure, Nancy Binkin

**Affiliations:** 1 Joint Doctoral Program in Public Health (Global Health), San Diego State University/University of California, San Diego, USA; 2 Division of Global Public Health, Department of Medicine, University of California, San Diego, La Jolla, CA, USA; 3 Ministry of Health and Social Welfare, Maseru, Lesotho; 4 HIV/AIDS Section, UNICEF New York, NY, USA; 5 UNICEF Lesotho, UNICEF, Maseru, Lesotho; 6 Program Implementation and Country Support Unit, Elizabeth Glaser Pediatric AIDS Foundation, Washington DC, USA; 7 Elizabeth Glaser Pediatric AIDS Foundation Lesotho, Maseru, Lesotho; 8 Graduate School of Public Health, San Diego State University, San Diego, CA, USA

**Keywords:** mother-to-child transmission of HIV, PMTCT, Lesotho, HIV/AIDS, co-packaging, Minimum PMTCT Package

## Abstract

**Introduction:**

Mother-to-child transmission of HIV can be reduced to<5% with appropriate antiretroviral medications. Such reductions depend on multiple health system encounters during antenatal care (ANC), delivery and breastfeeding; in countries with limited access to care, transmission remains high. In Lesotho, where 28% of women attending ANC are HIV positive but where geographic and other factors limit access to ANC and facility deliveries, a Minimum PMTCT Package was launched in 2007 as an alternative to the existing facility-based approach. Distributed at the first ANC visit, it packaged together all necessary pregnancy, delivery and early postnatal antiretroviral medications for mother and infant.

**Methods:**

To examine the availability, feasibility, acceptability and possible negative consequences of the Minimum PMTCT Package, data from a 2009 qualitative and quantitative study and a 2010 facility assessment were used. To examine the effects on ANC and facility-based delivery rates, a difference-in-differences analytic approach was applied to 2009 Demographic and Health Survey data for HIV-tested women who gave birth before and after Minimum PMTCT Package implementation.

**Results:**

The Minimum PMTCT Package was feasible and acceptable to providers and clients. Problems with test kit and medicine stock-outs occurred, and 46% of women did not receive the Minimum PMTCT Package until at least their second ANC visit. Providing adequate instruction on the use of multiple medications represented a challenge. The proportion of HIV-positive women delivering in facilities declined after Minimum PMTCT Package implementation, although it increased among HIV-negative women (difference-in-differences=14.5%, *p*=0.05). The mean number of ANC visits declined more among HIV-positive women than among HIV-negative women after implementation, though the difference was not statistically significant (*p*=0.09). Changes in the percentage of women receiving≥4 ANC visits did not differ between the two groups.

**Conclusions:**

If supply issues can be resolved and adequate client educational materials provided, take-away co-packages have the potential to increase access to PMTCT commodities in countries where women have limited access to health services. However, efforts must be made to carefully monitor potential changes in ANC visits and facility deliveries, and further evaluation of adherence, safety and effectiveness are needed.

## Introduction

Vertical transmission of HIV from mother to child during pregnancy, childbirth, and breastfeeding can be reduced to <5% using a strategy known as PMTCT (prevention of mother to child transmission), which includes the administration of a combination of antiretroviral medications (ARVs) during pregnancy, delivery, and for the duration of breastfeeding [1,2]. This approach, which was initially developed in the mid-1990s and has undergone subsequent modification and refinement, is highly cost-effective [3], and in most developed countries has resulted in the virtual elimination of vertical transmission [4].

PMTCT has been widely implemented in developing countries for a number of years, but it has not achieved the same levels of success as observed in the developed world. An important contributing factor has been the failure to achieve high coverage for each of the critical events in the PMTCT “cascade” of diagnosis and prophylaxis or treatment that are essential to successful prevention of transmission [5,6].

In many countries, a major obstacle to maintaining high coverage along the “cascade” is the confluence of the availability of facility-based PMTCT services and low utilization of antenatal care (ANC) and facility-based deliveries, both of which are essential for the initial diagnosis of HIV infection and administration of appropriate medications during pregnancy and delivery. The factors involved in low ANC and facility delivery coverage are complex and vary among countries but include the lack of trained personnel and facilities to deliver such services, limited geographic and financial access, and procurement and supply chain management weaknesses [7–11].

Lesotho, a landlocked country in southern Africa, is typical of many countries in the region, where rates of HIV are high and adequate delivery of PMTCT has been a challenge. It has the third highest HIV prevalence in the world [12], with an estimated adult HIV prevalence of 23%, and 28% prevalence among women seeking ANC [13]. The Government of Lesotho (GoL) initiated a PMTCT program in 2003 [14], resulting in an increase in the number of women given prophylaxis or treatment to prevent vertical transmission. However, like many countries, its progress was slower than expected and by 2007 less than half of identified HIV-exposed infants were receiving ARV prophylaxis [13,15].

Access to healthcare is a particularly important challenge in Lesotho, a mountainous country where over three-quarters of the population (77%) live in rural areas [16] and women often have to travel long distances over difficult terrain to seek care. The 2009 Demographic and Health Survey (DHS) found that 92% of women who had given birth in the preceding 5 years had received at least one ANC visit, but 30% did not receive the World Health Organization (WHO)—recommended four or more visits [17], and even more (41%) did not deliver in a health facility [18].

**Figure 1:**
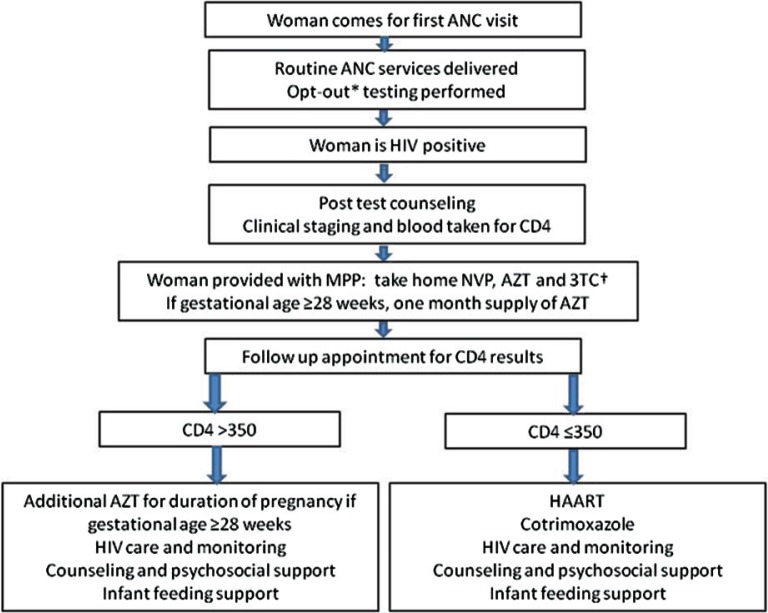
Minimum PMTCT Package algorithm, Lesotho.

The recognition that such attrition was a fundamental barrier to achieving the Ministry of Health and Social Welfare's (MOHSW) goal of universal access to PMTCT by 2011 led the GoL to introduce a co-package of medicines called the Minimum PMTCT Package (MPP) in 2007 [19]. This country-led initiative was designed to increase the opportunities for HIV-positive women who may have limited interactions with the health system to adhere to the 2006 WHO recommendations for PMTCT [1] by offering diagnostic and treatment services within the ANC clinics and providing HIV-positive pregnant women with a take-away collection of medicines to reduce the risk of vertical transmission. The medicines to be used at labour and delivery were provided at the time of diagnosis—generally the first ANC visit [19]. If the first ANC visit occurred at 28 weeks gestational age or more, women were also given a month's supply of antenatal ARV prophylaxis (AZT) until results of CD4 testing were obtained. Pills were packaged in bottles and envelopes and infant ARV suspensions were dispensed in foil-wrapped syringes; when available, clinics were instructed to put the individual items in a brown paper envelope. Women were encouraged to return for subsequent visits and to deliver in facilities, and if they did return, they were given additional AZT or started on HAART (highly active antiretroviral therapy) based on CD4 results. However, if they did not come back for further ANC or delivered at home, they had at least been given the appropriate medicines to greatly reduce the risk of HIV transmission during labour and delivery. Details of Lesotho's MPP diagnostic and treatment algorithm are shown in Figure 1.

*Indicates that HIV testing is standard procedure unless a patient expressly refuses testing.

†MPP contents: Intrapartum/7-day postpartum maternal ARVs (a tablet of NVP to be taken at the onset of labour and a supply of AZT and 3TC to be taken during labour and for seven days after delivery); 7-day or 28-day supply of postpartum infant ARVs (NVP suspension to be given at birth; AZT suspension, duration depended on duration of maternal antenatal ARVs)

Adapted from Lesotho's 2007 Guidelines for the Prevention of Mother to Child Transmission of HIV [19], regimens in line with WHO 2006 guidelines [1].

In 2007, the GoL tripled the number of facilities providing PMTCT. Concomitantly, the MPP distribution approach was rapidly scaled up in six of the country's 10 districts; rollout was nationwide by the end of 2008. GoL coverage statistics indicate that the number of women accessing ANC increased between 2007 and 2010, as did the number of women and infants receiving ARV prophylaxis and treatment [13]. Assessments conducted in 2009 and 2010 identified a number of obstacles to successful implementation and coverage improvement [20,21]. These challenges resulted in several important program adjustments and contributed to the development of a modified version of co-packaged medicines known as the Mother Baby Pack that the GoL has been implementing since January 2011.

Some countries have indicated an interest in implementing some form of take-away commodity package. The goal of our study was therefore to identify key learnings from Lesotho's experience with the Minimum PMTCT Package to inform future programming and evaluation of co-packaged medicines. Specifically, we examine what is known about the feasibility and acceptability of this approach to PMTCT, as well as unanticipated negative consequences. We also identify certain critical points and challenges encountered during the implementation and scale-up of the Lesotho co-packaging strategy that would need to be addressed and closely monitored to maximize its impact on preventing mother-to-child transmission of HIV.

## Methods

To better understand the rationale of the MPP and the required steps for its success, the authors reviewed international PMTCT recommendations [1,2,22,23] and national PMTCT guidelines for several sub-Saharan countries [19,24–27] and obtained input from staff that had been involved in the design and implementation of the MPP in Lesotho. A conceptual framework was then developed against which feasibility, acceptability, service uptake analyses, and potential detrimental effects could be examined. Based on this framework, a series of eight potential barriers for the successful functioning of the MPP was formulated, along with specific questions and data sources for each (Table 1).

**Table 1:** Conceptual framework for successful implementation of the Minimum PMTCT Package

Critical point	Question	Outcomes examined	Data source
1. The MPP should not interfere with ANC utilization: The MPP is designed to increase the likelihood that women who do not come for multiple ANC visits and/or do not deliver in a facility can prevent mother to child transmission of HIV. Once they have the MPP, women may be less motivated to come for subsequent ANC and obtain the recommended 4+ visits.	• Is the introduction of the MPP associated with changes in patterns of ANC utilization?	– Mean number of ANC visits among HIV-positive and HIV-negative women before and after MPP implementation – Percentage of HIV-positive and HIV-negative women receiving 4+ ANC visits before and after MPP implementation	DHS 2009
2. The MPP should be widely available: If the MPP is to have impact on a population basis, it must be widely available, especially in clinics serving remote areas where access to ANC and delivery facilities is lower.	• How many facilities are actually providing PMTCT/MPP, by type of facility and by district access to ANC and facility delivery?	– Percentage of facilities providing the MPP, stratified by district and facility type	2010 assessment (facilities providing MPP); DHS 2009 (% of women in district who got 4+ ANC visits and delivered in facility)
3. Integration, feasibility, and acceptability to providers: At facility level, successful delivery of the MPP requires that it be integrated into routine services for pregnant women, that those who deliver it are adequately trained, that it does not require excessive staff time to prepare and distribute, and that staff perceive an added benefit.	• To what extent was the MPP integrated in routine ANC activities? • How many facilities have at least one person trained in PMTCT? • Who assembled the MPP kits, and what burden did it pose on other activities? • What do healthcare workers think of the MPP?	– Percentage of sites where ANC and PMTCT provided in same physical location; where PMTCT information contained in ANC registry – Percentage of sites with at least one trained PMCT provider – Staff assembling MPP; time required to assemble it; interference with other activities – Staff opinions of MPP	2009 assessment (facility checklist, provider interviews) 2010 assessment (trained providers only)
4. The MPP requires on-site rapid testing and an uninterrupted supply of test kits and drugs: The success of the MPP is contingent on testing pregnant women for HIV on the first visit and providing them at that time with the MPP. Being able to obtain results of a CD4 count by the second visit is highly desirable, as is performing DBS collection for DNA PCR testing on-site to ensure that the mother and baby get optimal treatment. Finally, drugs should also not be wasted and should not be expired.	• During which ANC visit did women receive the MPP? • How many facilities that have provided PMTCT perform on site rapid testing, CD4, and collect DBS for PCR? • How many facilities that have provided PMTCT experienced stock-outs of test kits? • How many facilities had any stock-outs of critical MPP drugs, and how long did these stock-outs last? • Were there other supply and packaging issues? • What is done with unused drugs to reduce wastage and with expired drugs?	– ANC visit during which MPP received – Reported availability of testing – Percentage of facilities reporting stock-outs within past 3 or 6 months – Percentage experiencing stock-outs of any of the 5 medications in the MPP and reported duration – Answers to open-ended questions on supply issues, wastage, and expired drug disposal	2009 assessment (client and provider interviews, key informant interviews, and facility checklist) 2010 assessment
5. Clients should understand how to use the MPP and perceive its value in preventing vertical transmission: Clients should be accepting of the MPP and find it feasible to use in their personal circumstances. They need a good understanding of what drugs to use when	• Are women accepting of the MPP? • What problems do women encounter in using the MPP? • Did the clients receive oral AND either written or pictorial instructions on the use of the medications?	– Anecdotes from interviews – Percentage of women receiving written or pictorial instructions	2009 Assessment client interviews
6. The MPP should not adversely affect quality of ANC: Distribution of the MPP requires extra time and effort on the part of ANC providers. It should not negatively affect the quality of care received during antenatal care visits for women, regardless of their HIV status	• Is the introduction of the MPP associated with changes in the delivery of routine ANC interventions?	– Percentage of HIV-positive and HIV-negative women who receive information about signs of complications, have weight, and BP measured and urine and blood samples taken.	DHS 2009
7. Women should continue to deliver in facilities: Women who receive the MPP should still deliver in facilities given the high risks of HIV-positive women for poor pregnancy outcomes [4]. However, if facility-based delivery is not possible, the women should appropriately use the drugs in the MPP.	• Do women who receive the MPP still deliver in facilities?	– Percentage of HIV-positive and HIV-negative women who deliver in a facility, before and after MPP.	DHS 2009
8. Exposed infants should receive appropriate post-delivery care: PMTCT services are most effective as part of an overall continuum of care that includes quality ANC care, facility-based deliveries, and infant immunization uptake. Infants should be seen within the first six weeks of life for PCR testing and decisions about further medications.	• Is the introduction of the MPP associated with changes in first immunization visit?	– Percentage of children of HIV-positive and HIV-negative women receiving DPT1 at ≤3 months (proxy for DNA PCR test visit) before and after MPP	DHS 2009

### Data sources and analysis

#### The 2009 assessment

This MOHSW and UNICEF-commissioned study was designed to investigate the procurement and supply chain management of the MPP, its sociocultural acceptability, and the feasibility of scale-up. Methods are detailed in the study report [20], but briefly, data were collected in November–December 2009 using rapid, mixed-methods techniques that consisted of interviews with key informants from the MOHSW and national pharmacy services as well as other key partners; a facility checklist; interviews of service providers; and exit interviews of ANC, delivery, and postnatal visit patients. In addition, separate focus groups for men and women were conducted at community level to assess normative values regarding PMTCT and the MPP. The facility checklist and the service providers and patient interviews were conducted in 42 facilities: 17 of the 18 hospitals in the country, all five filter clinics (an interim level of service between a health centre and hospital), as well as 20 health centres that had been selected using district-level probability-proportionate-to-size sampling. The facility checklists, focus groups, and patient and staff interviewers were conducted by local teams, each of which included a nurse, and were hired and trained by the consulting firm that conducted the evaluation, with field supervision provided by the Lesotho MOHSW, WHO and the independent consulting firm. Patients were selected for exit interviews using a systematic random sampling frame. Data were available for a total of 150 providers and 214 patients, all of whom provided verbal consent [20]. Ethical approval was obtained from the Research and Ethics Committee of the Lesotho MOHSW.

Data from the assessment report was used where feasible. In addition, however, the original facility checklist data was re-analysed to better describe the extent to which facilities, particularly clinics, were adequately equipped to provide services for women who may have lacked access to subsequent ANC visits and facility delivery. Finally, the open-ended answers on the individual health worker and client questionnaires were examined to better understand their knowledge of and attitudes towards the MPP.

#### The 2010 assessment

This comprehensive facility HIV services assessment, which was funded by USAID and conducted by the Elizabeth Glaser Pediatric AIDS Foundation (EGPAF), was designed to evaluate the capacity of each health facility in Lesotho to fully implement comprehensive HIV/AIDS services and programs, including PMTCT. Data were collected in June 2010 using a structured questionnaire and chart data abstraction in each of Lesotho's 252 health facilities, of which 203 sites offered ANC services. In each facility, data were extracted from patient records and cards for the previous 3 months. Data collection was conducted by staff from EGPAF and the MOHSW who attended a one-day training course on correct survey procedures and data instruments use [21]. Ethical approval was obtained from the Research and Ethics Committee of the Lesotho MOHSW.

For the purpose of this analysis, findings from the report were examined; EGPAF also provided supplementary analyses of facility-level availability of care, testing capacities and medications.

#### The Lesotho 2009 Demographic and Health Survey

This nationally representative, individual level survey was conducted between October 2009 and January 2010, and was implemented by the MOHSW and Bureau of Statistics, with funding from USAID and technical support from Measure DHS. Two-stage cluster sampling was used to select households, and all women age 15–49 within a selected household were eligible to be interviewed. Interviews and HIV testing were conducted by enumerators who underwent extensive training on the DHS protocols and procedures [18]. A total of 7624 women aged 15–49 were interviewed using structured questionnaires on personal data assistants, with an eligible woman response rate of 98% [18]. HIV testing was conducted in 50% of the households where women were interviewed (*n*=4016). Testing was anonymous and was performed on dried blood spots collected at the time of the interview.

For these analyses, records from the individual questionnaires were linked with the HIV test results for all women who had received a DHS-administered HIV test and had given birth during 2005–2009 (*n*=1545). For women with more than one birth during this period, only the most recent birth was used. Women who were HIV-tested were not significantly different from untested women in terms of urban/rural residence, marital status, age, education level, literacy, wealth, parity and years of residency (results not shown).

The linked DHS data were used to investigate the effect of the MPP implementation on maternal and child health services and service utilization. Specific outcomes examined were≥4 ANC visits, the mean number of ANC visits, the quality of ANC care, facility deliveries, and whether or not the infant was brought into a facility by 3 months of age for DPT1 (a proxy for the recommended 6-week follow-up visit for DNA PCR testing). Women were considered to have received quality care if they reported all of the following during their most recent pregnancy: being informed of signs of pregnancy complications; being weighed; having blood pressure, urine and blood samples taken.

A difference-in-differences analytic approach was used to assess the percentage change in each outcome after MPP implementation. Specifically, self-reported service utilization from before and after the MPP rollout was compared between women who should have received the MPP (HIV-positive women) and those that should not have received it (HIV-negative women). The HIV-negative group provided the background trends in ANC and delivery services against which the experience of the HIV-positive women could be compared. Pre-/post-implementation of the MPP was differentially defined by district, as the program was implemented on a rolling basis. For purposes of the analysis, the pre-implementation period was 2005–2006 and post was 2007–2009 for Butha Buthe, Leribe, Berea, Mafeteng, Maseru and Mohale's Hoek. For the remaining districts of Quthing, Quacha's Nek, Mokhotlong and Thaba-Tseka, the corresponding years for analysis were 2005–2007 and 2008–2009.

The logit models used for this approach contained demographic covariates independently associated with HIV status as well as the outcome in question (assessed by Rao-Scott F-adjusted Chi-square tests [categorical variables] or *t*-tests [continuous variables] with a 0.05 significance cut-off) as well as HIV status and a pre-/post-implementation dummy variable which were interacted to create the difference-in-difference estimator. District of residence was also included to adjust for district-specific factors not accounted for in other demographic variables, as well as possible spillover effects. No multi-collinearity between variables in any model was detected using a tolerance level of 0.10. Marginal effects at the mean were then applied to the specified logit models to calculate model adjusted predicted prevalence before and after MPP implementation in HIV-positive and HIV-negative women (HIV positive, pre-implementation [*n*=149]; HIV positive, post-implementation [*n*=250]; HIV negative, pre-implementation [*n*=381]; HIV negative, post-implementation [*n*=765]). All analyses with DHS data were weighted and adjusted for the complex survey design [28].

#### Additional data sources

Data from the 2010 and 2011 Annual Joint Reports of the MOHSW [13,29] and the 2010 UNGASS Report on the Status of the National Response to the 2001 Declaration of Commitment on HIV and AIDS [30] were examined for further historical background and information on PMTCT trends.

All statistical calculations were conducted using SAS v.9.2 and Stata 10.

## Results

Eight components critical to the successful prevention of mother to child transmission of HIV with the MPP were identified by reviewing the conceptual framework of the program (Table 1). The following presents the findings for each critical point.

### 

#### 1. The MPP should not interfere with ANC utilization

Analysis of 2009 DHS data showed that both HIV-positive and HIV-negative women came for their first ANC visit at approximately 4.4 months gestational age. The proportion of women who attended at least four ANC visits declined both HIV positive and HIV negative between the pre- and post-implementation periods, but this was not significantly different between the two groups (Table 2). There was a concomitant decline in the adjusted number of ANC visits which was greater in HIV-positive women than in HIV-negative women, though this difference did not reach statistical significance (*p*=0.09; Table 2).

**Table 2:** Adjusted prevalence of select outcomes in HIV-positive and HIV-negative women in Lesotho from DHS 2009 data

Outcome	HIV status	Pre-implementation % (95% CI)	Post-implementation % (95% CI)	Difference in differences % (*p*-value^z^)
≥4 ANC visits^y^				
	HIV positive	75.0 (66.0–82.3)	62.5 (54.9–69.6)	−1.4 (0.92)
	HIV negative	77.6 (72.4–82.0)	66.5 (62.4–70.4)	
Mean number of ANC visits^x^				
	HIV positive	5.2 (4.7–5.8)	4.3 (3.9–4.7)	−0.6 (0.09)
	HIV negative	5.0 (4.7–5.2)	4.7 (4.5–4.9)	
Quality of ANC^w^				
	HIV positive	26.4 (18.8–35.8)	30.2 (23.9–37.4)	−4.3 (0.56)
	HIV negative	26.8 (21.2–33.4)	34.9 (30.4–39.6)	
Facility deliveries^v^				
	HIV positive	57.7 (47.0–67.8)	48.9 (41.2–56.5)	−14.5 (0.05)
	HIV negative	55.9 (49.0–62.5)	61.5 (56.9–65.9)	
DPT1 within 3 months of birth^u^				
	HIV positive	86.6 (75.5–93.2)	91.4 (85.5–95.1)	2.2 (0.84)
	HIV negative	91.4 (86.2–94.7)	94.0 (91.0–96.0)	

z *t*-Test on difference in difference estimator.

y Adjusted for marital status, rural/urban residence, wealth quintile, number of children and district of residence.

x Values shown are means, not percentages. Adjusted for rural/urban residence, wealth quintile, number of children and district of residence.

w Composite indicator representing women who reported being informed of signs of pregnancy complications, and being weighed, and having blood pressure, urine and blood samples taken. Denominator is women who attended ≥1 ANC visit. Adjusted for rural/urban residence, maternal age, wealth quintile and district of residence.

v Adjusted for marital status, rural/urban residence, maternal age, wealth quintile, number of children and district of residence.

u Adjusted for wealth quintile, number of children and district of residence.

#### 2. The MPP should be widely available

The 2010 assessment demonstrated that within three years of the MPP program initiation and approximately one year after full national scale-up, four out of five (81%) facilities (including 79% of health centres) were providing the MPP within the context of ANC. While all but one hospital in the country provided the MPP, it was available in only 88% of health centres [21]. The majority (80%) of facilities held ANC clinics only once a week [21]. MPP coverage was not uniform across districts and ranged from 69% to 100%. Based on a secondary analysis combining data from the 2009 DHS and 2010 assessment, the percentage of facilities providing the MPP tended to be lower in several of the districts where the vast majority of pregnant women lived in rural areas (data not shown).

#### 3. The MPP should be integrated, feasible and acceptable to providers

According to the 2009 assessment, PMTCT services were offered in the same room and on the same days as ANC services in most (79%) of the health facilities surveyed; in 2/3 (67%), the daily records of PMTCT clients were maintained in the same register as all ANC and postnatal care clients [20]. The 2010 assessment demonstrated that 87% of facilities, including 86% of health centres, had at least one nurse with PMTCT training (EGPAF, personal communication).

In terms of burden, the 2009 assessment revealed that nursing staff, which included nursing assistants, were responsible for assembling the packs in most locations; pharmacists or pharmacy technicians were not involved in any cases. The burden of assembling the packs appeared relatively minor; only one in four stated it took 15 minutes or more, while many reported that it took five minutes or less. Knowledge of the handling of expired medicines was not optimal [20].

Although almost half (48%) of the providers stated that assembly of the kit took time away from other activities [20], they seemed to have a positive attitude toward the MPP. In the 2009 assessment, many specifically expressed that they felt it saved lives and that it was reducing the number of babies born HIV positive. However, some questioned the patients’ ability to follow the complex instructions. Providers also expressed concern about adherence issues in women who had not disclosed their HIV status to their partners.

#### 4. The MPP requires on-site rapid testing and an uninterrupted supply of test kits and medicines

All facilities that distributed the MPP performed rapid HIV testing on site. With respect to CD4 testing, which was needed to determine whether women should be initiated onto HAART or be given prophylactic AZT, nearly half of the facilities (47%) reported performing CD4 tests for pregnant women on-site, although there was a high variability in the distribution of this indicator across districts. In most facilities that did have testing capacities, CD4 tests were performed only once a week [21].

Many facilities experienced problems with the availability of rapid test kits, CD4 testing materials and dried blood spot test kits. In the 2009 assessment, approximately 40% of clinics and 60% of hospitals had had stock-outs of rapid test kits in the past 6 months, for example, with a number of these stock-outs lasting for a period of 2 months or more [20]. According to the more recent 2010 assessment, 40% of facilities experienced a stock-out of at least one test kit supply (including 22% who experienced rapid test kit stock-outs) during the last three months, with values for the districts ranging from 16% to 68% [21].

Major problems were also encountered with medicine stock-outs and, in some cases, with medicine expiration. In the 2009 assessment, only a minority (44%) of the facilities reported no medicine stock-outs in the past 6 months, and more than a quarter experienced stock-outs or expired stocks of two or more of the medicines, most commonly AZT and the paediatric suspensions of nevirapine and AZT. In some instances the absence of these medicines lasted a matter of days, but in others, it lasted for months [20]. In the 2010 assessment, when asked about stock-outs in the past 3 months, 32% of facilities reported stock-outs of at least one medicine (EGPAF, personal communication). There were also problems with stock-outs of capped syringes and packaging materials for the paediatric suspension syringes. When stock-outs occurred, the national drug supply system tried to organize re-distribution from other sites, but in other cases, either the patients had to be referred to other facilities or they were not been given the pack or they had been given insufficient packs [20].

The combined effect of testing availability and medicine stock-outs produced some sobering results, as shown in Table 3, which is based on data from the 2010 assessment. Overall, of the 143 health centres providing the MPP, 111 (78%) consistently had all the elements in place to effectively deliver the package (EGPAF, personal communication).

**Table 3:** Number and percentage of health centres experiencing stock-outs within the past 3 months of critical test kits and drugs required for successful MPP delivery among all health centres and among those providing the MPP, Lesotho, 2010

Critical component	*N*	Percentage of all health centres	Percentage of the health centres that provided MPP
Provides ANC	203	100	–
Provides MPP	143	70	100
AND rapid test on site	140	69	98
AND no stock-outs of AZT	130	64	91
AND no stock-outs of nevirapine	127	63	89
AND no stock-outs of Combivir	122	60	85
AND no stock-outs of paediatric suspension	111	55	78

The denominator for each row consists of the facilities that had the critical components in the rows above. Source: EGPAF, personal communication; based on 2010 assessment data.

Given the various stock-outs of testing reagents and medicine, only about half (54%) of the women interviewed in the 2009 assessment reported receiving the MPP on their first visit, an additional 22% on the second visit, but nearly a quarter did not receive it until at least the third visit [20]. As many women also sought ANC relatively late, 60% of women reported receiving their pack in month 7–9 of their pregnancy, with an additional 27% receiving it between 4 and 6 months and a relatively small minority in the first trimester.

Key informants identified drug availability as a major bottleneck to expansion. It was not clear the extent to which the stock-outs reflected poor drug management and ordering procedures or a rapid increase in demand, especially since in 2008, when Lesotho began providing outpatient health services free of charge and patient volume outstripped supplies. Another problem was the complexity of ordering the many components for local assembly of the MPP since different components were used up at different rates.

#### 5. Clients should understand how to use the MPP and perceive its value in preventing vertical transmission

Although materials were available in many sites on PMTCT in general and MPP-specific patient materials were later developed (EGPAF, personal communication), the 2009 survey found that there were no MPP-specific IEC or written instructions that could be taken home. Some facilities had prepared ad hoc materials, but for the most part instructions were provided orally, with instructions on when and how to administer the medicines written on the individual envelopes or containers.

In the 2009 client survey, most women reported that they understood all the instructions, but some of the women did express concern about the complexity of the instructions and the impatience of staff explaining them. Also some of the interviewed nurses expressed doubts about whether the women truly understood what had been explained and whether they could handle the medications [20].

Women were asked in the 2009 assessment to repeat the instructions they had been given about the MPP medicines. Only a small number of women described the need to administer the nevirapine suspension at birth and even fewer mentioned the administration of medications to the baby during the first week of life.

Interviews from the 2009 study demonstrated that some of the women who had been recently diagnosed HIV positive reported that they were worried and apprehensive about the MPP, but a large majority was very happy to have medication to prevent their child from getting sick. There was virtually no mention, however, of the convenience of having everything they needed.

The women who expressed most concern about the pack and being able to adhere were those who had not disclosed their HIV status to family members [20]. In addition to the concerns about disclosure, some women were particularly worried about the administration of nevirapine during delivery if they did not deliver in a facility and how this would lead others in the community to finding out their status. However, among women who delivered at home, about half said they had the birth attendant administer the medications, while others reported alternative solutions such as administering the medications themselves; only 10% of respondents stated that the baby did not get the medications [20].

#### 6. The MPP should not adversely affect the quality of ANC

The percentage of women who reported receiving all five components of quality ANC care from pre-implementation to post-implementation increased for both HIV-positive and HIV-negative women. While this trend was more pronounced among HIV-negative women, there were no significant differences between the two groups attributable to the implementation of the MPP (*p*=0.56; Table 2).

#### 7. Women should continue to deliver in facilities

HIV-positive and HIV-negative women had substantially different experiences with respect to facility deliveries. There was a 9% decrease in facility deliveries from pre-implementation to post-implementation for HIV-positive women, compared to a more than 5% increase for HIV-negative women (Table 2). The difference in differences was thus 14.5%, which was significant at the *p*<0.05 level.

#### 8. Exposed infants should receive appropriate post-delivery routine care

DPT1 coverage within 3 months of birth increased for the infants of both HIV-positive and HIV-negative women. The increase was greater in HIV-positive women (4.8% compared to 2.6%), though the overall difference in differences was not significant (*p*=0.84; Table 2).

## Discussion

The MPP was developed in Lesotho and implemented from 2007 to 2010 as an innovative response to the problem of limited health encounters during pregnancy and low rates of facility delivery. Within two years of program implementation, approximately 50% of the women served by facilities providing the MPP received it on their first ANC visit, thereby increasing the likelihood that they had the drugs necessary to prevent vertical transmission of HIV even if they did not subsequently seek facility-based care. This rapid scale-up, however, was accompanied by a statistically significant decline in facility-based deliveries among HIV-positive women as compared with the increase seen among HIV-negative women.

This study has numerous methodological limitations, including the use of retrospective assessments and survey data for our subsequent secondary analyses, and the lack of data to estimate the ultimate effectiveness of the MPP in preventing mother-to-child transmission. An inherent limitation of the information presented on client exit interviews is the de-facto restriction to women who sought facility-based care in the first place, a sample that is not representative of all women and most likely represents a best-case scenario. In addition, HIV test results from the 2009 DHS survey were used as a means of distinguishing HIV-positive from HIV-negative women. These test results do not necessarily indicate that a woman was HIV positive during her last pregnancy, or if she was, that she knew her status. Additionally, the assumption in the DHS analyses that all women living in the rollout districts would have had MPP access is flawed. Furthermore, since the DHS is based on self-reports, recall for outcomes such as number of ANC visits or quality of care during pregnancy may differ between women whose most recent pregnancy was closer to 2005 compared to women who had more recent pregnancies. Finally, the nature of data available also precluded investigation of the critical questions of retention, adherence [5,31–33], safety [34], resistance [35], effect on routine ANC and facility delivery utilization, or effectiveness in preventing vertical transmission.

Despite these limitations, our findings nonetheless suggest that several of the potential barriers to the successful prevention of mother-to-child transmission of HIV using co-packaged PMTCT medicines can be overcome. This analysis demonstrated: that (1) the approach of supplying co-packaged take-away medicines could be rapidly scaled up; (2) that it was feasible to deliver the MPP within the context of routine ANC; (3) that it was acceptable to the vast majority of providers and clients, both of whom perceived it as a potentially life-saving intervention; (4) that its implementation did not appear to adversely affect the quality of ANC; and (5) that it did not affect early access to care among exposed infants in the first months of life.

At the same time, however, a number of barriers were not fully overcome and should be taken into consideration by countries wishing to implement similar take-away co-packaging of PMTCT medicines. These include: (1) ensuring that it is available in remote areas where the need may be the greatest; (2) ensuring that women continue ANC and deliver in facilities even though they already have the medications needed to prevent HIV transmission to their infants; (3) providing on-site testing and an uninterrupted supply of test kits and medicines; and (4) enhancing client understanding of the timing and use of the multiple medications included in the pack.

Several key lessons emerged from this analysis that can better inform future programming involving PMTCT and co-packaged medicines., Although Lesotho managed to rapidly scale up the MPP in all districts of the country, some of the most rural districts that might have benefited the most actually had the lowest facility coverage of the MPP. In terms of coverage and equity, consideration should be given to identifying those areas in greatest need on the basis of remote location and current coverage and prioritizing these areas for scale-up.

With respect to ANC and facility delivery coverage, data from the DHS survey suggested a statistically significant decrease in facility deliveries among HIV-positive women as compared with a concomitant increase among HIV-negative women, even after adjusting for a wide variety of demographic and geographic factors. Although they did not reach statistical significance, there were also differences with respect to the number of ANC visits and completion of the recommended four or more visits. The role of the MPP in these findings is unclear since underlying factors such as the introduction of provider-initiated HIV testing polices for pregnant women or stigma also may have influenced HIV-positive women to utilize services differently than HIV-negative women. Nonetheless, these results are of concern and highlight the importance of on-going monitoring of potential declines in ANC attendance and facility deliveries, as well as further investigations into the reasons behind these declines. Additionally, these findings suggest that IEC activities should emphasize the importance of continuing ANC and delivering in a facility, and that community support structures to encourage facility-based service retention may be needed.

The availability of test kits and medications was a major issue, as it is in many health programs in developing country settings. If co-packaged medicines are meant to be distributed with a minimum of health system encounters, the uninterrupted availability of test kits and medicines is of even greater importance than in programs that assume women will be seen multiple times during pregnancy; the stock-out of any single element means that additional encounters will be needed. Take-away PMTCT prophylaxis programs will clearly require an organized and efficient monitoring and supply system to minimize stock-outs.

The Lesotho experience also underlined that adequate client understanding is a potentially serious barrier to the successful implementation of a take-away co-package. Any take-away pack will be complex and contain multiple medications to be administered to the mother and infant at different times. Communication strategies must be developed which incorporate written materials or inserts, facility based counselling, peer-to-peer support and other channels to ensure mothers learn and retain the information for use. Performing detailed field testing of communication methods, including package inserts, will be critical to its success.

Following the experiences of the Minimum PMTCT Package, in 2011, Lesotho launched the Mother Baby Pack, which, unlike the MPP, is distributed to all pregnant women. There are now three packs: one for HIV-negative women that contain vitamins, micronutrients and iron, and two different packs of ARV medicines based on CD4 count for women who are HIV positive, as per Option A of the 2010 WHO Guidelines [2]; the latter packs also contain the items included in the pack for HIV-negative women [13,25]. Common features of the packs include central packaging with on-site adjustment, preparation and testing of more comprehensive IEC materials for patients, and healthcare worker training. As was the case with the MPP, the current Mother Baby Pack does not cover the complete postnatal period, and is not offered as an alternative to facility-based services, but rather as a mechanism to ensure that women who find themselves unable to return for facility-based services have the medicines necessary to improve both their own health and that of their infant. Women are asked to bring their packs back at each visit, where they are checked and re-stocked as necessary by the healthcare workers. As part of this strategy, community health workers are following up those women who miss appointments to bring them back into the system, and encourage facility-based delivery. A formal evaluation of the Mother Baby Pack, which will address some of the questions raised by the MPP experience, is currently under way.

The Lesotho MPP was implemented in an era when the primary recommendation for PMTCT prophylaxis was AZT beginning at 28 weeks and ending 1 week after birth, paired with additional medications for the mother and infant during labour/delivery and postpartum [1]. This guidance has evolved, and the most recent WHO programmatic update highlights the greater simplicity and programmatic advantages of short-course (Option B) and lifelong (Option B+) antiretroviral therapy for pregnant women living with HIV [2,36]. This shift towards the use of fixed dose triple ARV regimens for both treatment and prevention of mother to child transmission of HIV will enable greater streamlining of co-packaged medicines. Simultaneously, it should remain a priority to establish effective monitoring, and to evaluate the impact of this approach on retention in facility-based care, as well as its overall safety and effectiveness.

## Conclusions

Lesotho's Minimum PMTCT Package proved both feasible and acceptable to providers and clients as a means of offering critical PMTCT drugs to HIV-positive women who face barriers to regular contact with the health system. There are numerous challenges to this approach, including supply chain interruptions, logistical difficulties, the dissemination of comprehensible instructions, and the possibility of actually decreasing a woman's likelihood of seeking facility-based care. The potential benefit is also substantial. Co-packaged medicines represent a novel programmatic approach that merits further evaluation of safety and effectiveness in the on-going fight to prevent and eliminate vertical HIV transmission.

## References

[1] World Health Organization (2006). Antiretroviral drugs for treating pregnant women and preventing HIV infection in infants: towards universal access: recommendations for a public health approach—2006 version.

[2] World Health Organization (2010). Antiretroviral drugs for treating pregnant women and preventing HIV infection in infants: recommendations for a public health approach—2010 version.

[3] Johri M, Ako-Arrey D (2011). The cost-effectiveness of preventing mother-to-child transmission of HIV in low- and middle-income countries: systematic review. Cost Eff Resour Alloc.

[4] Mepham SO, Bland RM, Newell ML (2011). Prevention of mother-to-child transmission of HIV in resource-rich and -poor settings. BJOG.

[5] Stringer JS, Sinkala M, Maclean CC, Levy J, Kankasa C, Degroot A (2005). Effectiveness of a city-wide program to prevent mother-to-child HIV transmission in Lusaka, Zambia. AIDS.

[6] Marcos Y, Ryan Phelps B, Bachman G (2012). Community strategies that improve care and retention along the prevention of mother-to-child transmission of HIV cascade: a review. J Int AIDS Soc.

[7] Kasenga F, Byass P, Emmelin M, Hurtig AK (2009). The implications of policy changes on the uptake of a PMTCT programme in rural Malawi: first three years of experience. Glob Health Action.

[8] Hodgkin D (1996). Household characteristics affecting where mothers deliver in rural Kenya. Health Econ.

[9] Parkhurst JO, Rahman SA, Ssengooba F (2006). Overcoming access barriers for facility-based delivery in low-income settings: insights from Bangladesh and Uganda. J Health Popul Nutr.

[10] Myer L, Harrison A (2003). Why do women seek antenatal care late? Perspectives from rural South Africa. J Midwifery Womens Health.

[11] Sprague C, Chersich MF, Black V (2011). Health system weaknesses constrain access to PMTCT and maternal HIV services in South Africa: a qualitative enquiry. AIDS Res Ther.

[12] AIDSInfo [database on the Internet] (2011). UNAIDS. http://www.aidsinfoonline.org/.

[13] Government of Lesotho (2011). Annual Joint Review Report 2010/11 FY.

[14] Government of Lesotho Ministry of Health & Social Welfare (2007). Prevention of mother to child transmission of HIV and paediatric HIV care and treatment: scale up plan, 2007/08–2010/11.

[15] Government of Lesotho (2008). Annual Joint Review Report 2007/08 FY.

[16] Government of Lesotho (2009). 2006 Lesotho Population and Housing Census.

[17] World Health Organization (2002). WHO antenatal care randomized trial: manual for the implementation of the new model.

[18] Ministry of Health and Social Welfare (MOHSW) [Lesotho], ICF Macro (2010). Lesotho Demographic and Health Survey 2009.

[19] Government of Lesotho (2007). Guidelines for the prevention of mother to child transmission of HIV (PMTCT).

[20] Masazi Development Associates (2010). Assessment of the Minimum PMTCT Package (MPP) Lesotho.

[21] Elizabeth Glaser Pediatric AIDS Foundation (2010). HIV/AIDS service provision in Lesotho: a baseline report.

[22] World Health Organization (2010). PMTCT strategic vision 2010–2015: preventing mother-to-child transmission of HIV to reach the UNGASS and Millennium Development Goals.

[23] Inter-Agency Task Team on Prevention of HIV Infection in Pregnant Women, Mothers and their Children (2007). Guidance on global scale-up of the prevention of mother to child transmission of HIV: towards universal access for women, infants and young children and eliminating HIV and AIDS among children.

[24] Government of Malawi Ministry of Health (2008). Prevention of mother to child transmission of HIV and paediatric HIV care guidelines. Lilongwe: GoM.

[25] Government of Lesotho (2010). National guidelines for the prevention of mother to child transmission of HIV.

[26] National Department of Health, South Africa, South African National AIDS Council (2010). Clinical guidelines: PMTCT (Prevention of Mother-to-Child Transmission).

[27] Ministry of Health, Republic of Kenya (2009). Guidelines for Prevention of Mother to Child Transmission (PMTCT) of HIV/AIDS in Kenya.

[28] Rutstein S, Rojas G (2006). Guide to DHS statistics.

[29] Government of Lesotho (2010). Annual Joint Review Report 2009/10 FY.

[30] National AIDS Commission, Government of Lesotho (2010). Lesotho UNGASS country report: status of the national response to the 2001 declaration of commitment on HIV and AIDS.

[31] Kirsten I, Sewangi J, Kunz A, Dugange F, Ziske J, Jordan-Harder B (2011). Adherence to combination prophylaxis for prevention of mother-to-child-transmission of HIV in Tanzania. PLoS One.

[32] Delvaux T, Elul B, Ndagije F, Munyana E, Roberfroid D, Asiimwe A (2009). Determinants of nonadherence to a single-dose nevirapine regimen for the prevention of mother-to-child HIV transmission in Rwanda. J Acquir Immune Defic Syndr.

[33] Kuonza LR, Tshuma CD, Shambira GN, Tshimanga M (2010). Non-adherence to the single dose nevirapine regimen for the prevention of mother-to-child transmission of HIV in Bindura town, Zimbabwe: a cross-sectional analytic study. BMC Public Health.

[34] Coffie PA, Ekouevi DK, Chaix ML, Tonwe-Gold B, Clarisse AB, Becquet R (2008). Maternal 12-month response to antiretroviral therapy following prevention of mother-to-child transmission of HIV type 1, Ivory Coast, 2003–2006. Clin Infect Dis.

[35] Coovadia A, Abrams EJ, Stehlau R, Meyers T, Martens L, Sherman G (2010). Reuse of nevirapine in exposed HIV-infected children after protease inhibitor-based viral suppression: a randomized controlled trial. JAMA.

[36] World Health Organization (2012). Programmatic update: use of antiretroviral drugs for treating pregnant women and preventing HIV infection in infants. Executive summary.

